# Serum cystatin C concentration as a marker of acute renal dysfunction in critically ill patients

**DOI:** 10.1186/cc3044

**Published:** 2005-02-07

**Authors:** Patricia Villa, Manuel Jiménez, Maria-Cruz Soriano, Jesus Manzanares, Pilar Casasnovas

**Affiliations:** 1 Intensive Care Unit, Hospital Universitario La Paz, Madrid, Spain; 2 Biochemistry Unit, Hospital Universitario La Paz, Madrid, Spain

## Abstract

**Introduction:**

In critically ill patients sudden changes in glomerular filtration rate (GFR) are not instantly followed by parallel changes in serum creatinine. The aim of the present study was to analyze the utility of serum cystatin C as a marker of renal function in these patients.

**Methods:**

Serum creatinine, serum cystatin C and 24-hour creatinine clearance (C_Cr_) were determined in 50 critically ill patients (age 21–86 years; mean Acute Physiology and Chronic Health Evaluation II score 20 ± 9). They did not have chronic renal failure but were at risk for developing renal dysfunction. Serum cystatin C was measured using particle enhanced immunonephelometry. Twenty-four-hour body surface adjusted C_Cr _was used as a control because it is the 'gold standard' for determining GFR.

**Results:**

Serum creatinine, serum cystatin C and C_Cr _(mean ± standard deviation [range]) were 1.00 ± 0.85 mg/dl (0.40–5.61 mg/dl), 1.19 ± 0.79 mg/l (0.49–4.70 mg/l), and 92.74 ± 52.74 ml/min per 1.73 m^2 ^(8.17–233.21 ml/min per 1.73 m^2^), respectively. Our data showed that serum cystatin C correlated better with GFR than did creatinine (1/cystatin C versus C_Cr_: r = 0.832, *P *< 0.001; 1/creatinine versus C_Cr_: r = 0.426, *P *= 0.002). Cystatin C was diagnostically superior to creatinine (area under the curve [AUC] for cystatin C 0.927, 95% confidence interval 86.1–99.4; AUC for creatinine 0.694, 95% confidence interval 54.1–84.6). Half of the patients had acute renal dysfunction. Only five (20%) of these 25 patients had elevated serum creatinine, whereas 76% had elevated serum cystatin C levels (*P *= 0.032).

**Conclusion:**

Cystatin C is an accurate marker of subtle changes in GFR, and it may be superior to creatinine when assessing this parameter in clinical practice in critically ill patients.

## Introduction

Glomerular filtration rate (GFR) is considered the best marker of renal function, and serum creatinine is the most commonly used biochemical parameter to estimate GFR in routine practice. However, there are some shortcomings to the use of this parameter. Factors such as muscle mass and protein intake can influence serum creatinine, leading to an inaccurate estimation of GFR. Normal serum creatinine may be observed in individuals with significantly impaired GFR [1,2]. Moreover, in unstable, critically ill patients, acute changes in renal function can make real-time evaluation of GFR using serum creatinine difficult.

Cystatin C is a nonglycosylated protein that belongs to the cysteine protease inhibitors, cystatin superfamily [3]. These proteins play an important role in the regulation of proteolytic damage to the cysteine proteases. Cystatin C is produced at a constant rate by nucleated cells [4]. It is found in relatively high concentrations in many body fluids, especially in the seminal fluid, cerebrospinal fluid and synovial fluid [5]. Its low molecular weight (13.3 kDa) and positive charge at physiological pH levels facilitate its glomerular filtration. Subsequently, it is reabsorbed and almost completely catabolized in the proximal renal tubule [6,7]. Therefore, because of its constant rate of production, its serum concentration is determined by glomerular filtration [8-11]. Moreover, its concentration is not influenced by infections, liver diseases, or inflammatory diseases. Use of serum cystatin C as a marker of GFR is well documented, and some authors have suggested that it may be more accurate than serum creatinine for this purpose [12-19].

The difficulties associated with monitoring and evaluating GFR in critically ill individuals are well known. Thus far no studies evaluating serum cystatin C as a marker of GFR in these patients have been reported. The aim of the present study were to determine the accuracy of serum cystatin C concentration as a marker of GFR in critically ill individuals.

## Methods

Fifty patients, aged 21–86 years (mean 54 years), who were admitted to the intensive care unit at the Hospital Universitario La Paz in Madrid, Spain between January and September 2001, were included in the study. All patients were at risk for developing renal failure (haemodynamically unstable patients, septic patients, individuals receiving nephrotoxic drugs and others). Patients receiving corticoid therapy or with thyroid diseases were excluded. The patients' demographic characteristics and clinical conditions are summarized in Table 1.

A serum sample was drawn from each patient in the morning (between 07:00 and 10:00) to determine serum creatinine and serum cystatin C. A 24-hour urine sample was obtained just before the serum sample to calculate the creatinine clearance (C_Cr_) using the following formula: C_Cr _(ml/min) = (urine volume × urine creatinine)/(serum creatinine × 1440).

Serum creatinine values were obtained according to standard laboratory methods. C_Cr _was adjusted to body surface (ml/min per 1.73 m^2^). Cystatin C values were obtained using particle enhanced immunonephelometry [10]. Normal serum creatinine values range from 0.6 to 1.3 mg/dl, and normal serum cystatin C values range from 0.6 to 1 mg/l. Renal dysfunction was defined as C_Cr _below 80 ml/min per 1.73 m^2^.

### Statistical analysis

The data are expressed in mean ± standard deviation (range). Correlations between quantitative data were determined using Pearson's test. *P *< 0.05 was considered statistically significant. The diagnostic value of serum cystatin C and serum creatinine for identifying renal dysfunction was evaluated using receiver operating characteristic curve analysis, and the data are expressed as area under the curve (AUC; 95% confidence interval). For statistical analysis, the SPSS R 9.0 (SPSS Inc., Chicago, IL, USA) program was used.

## Results

The mean serum creatinine concentration was 1.00 ± 0.85 mg/dl (0.40–5.61 mg/dl) and the mean serum cystatin C concentration was 1.19 ± 0.79 mg/l (0.49–4.70 mg/l). The mean C_Cr _adjusted for the body surface was 92.74 ± 52.74 ml/min per 1.73 m^2 ^(8.17–233.21 ml/min per 1.73 m^2^).

**Figure 1:**
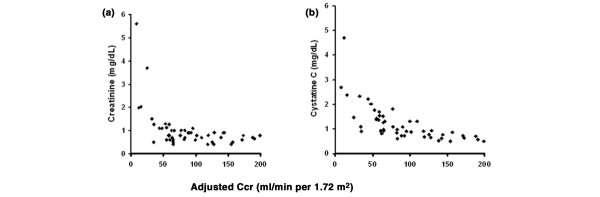
Relationships of **(a) **serum creatinine and **(b) **serum cystatin C to creatinine clearance (C_Cr_).

**Figure 2:**
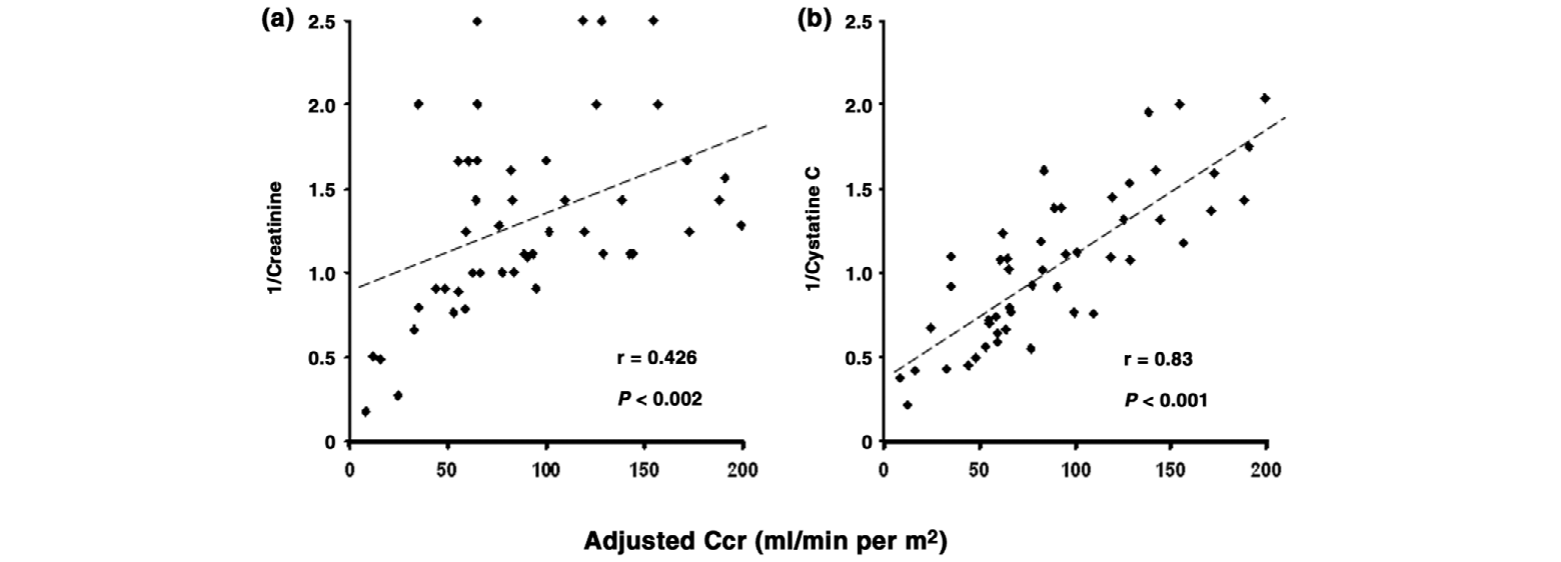
The **(a) **inverse of serum creatinine (1/creatinine) and the **(b) **inverse of cystatin C (1/cystatin c) were plotted against creatinine clearance (C_Cr_) for each of patient (*P *= 0.002 versus *P *< 0.001).

A decline in C_Cr _was followed by an increase in levels of serum creatinine and serum cystatin C (Fig. 1). The inverse of the serum cystatin C and serum creatinine levels were plotted against C_Cr _to determine the relationships of those parameters to this marker of renal function (Fig. 2a,b). There were significant correlations between C_Cr _and 1/serum creatinine (r = 0.426, *P *= 0.002) and between C_Cr _and 1/serum cystatin C (r = 0.832, *P *< 0.001).

Twenty-five out of the 50 patients enrolled in the study had renal dysfunction (C_Cr _<80 ml/min per 1.73 m^2^). Five (20%) of these 25 patients with renal dysfunction had elevated serum creatinine concentrations, whereas 19 (76%) of them had elevated serum cystatin C levels at the time of renal dysfunction (*P *= 0.032). On the other hand, serum creatinine levels were within normal ranges in all patients with normal C_Cr _(>80 ml/min per 1.73 m^2^) whereas 23 (92%) of them had normal concentrations of serum cystatin C.

**Figure 3:**
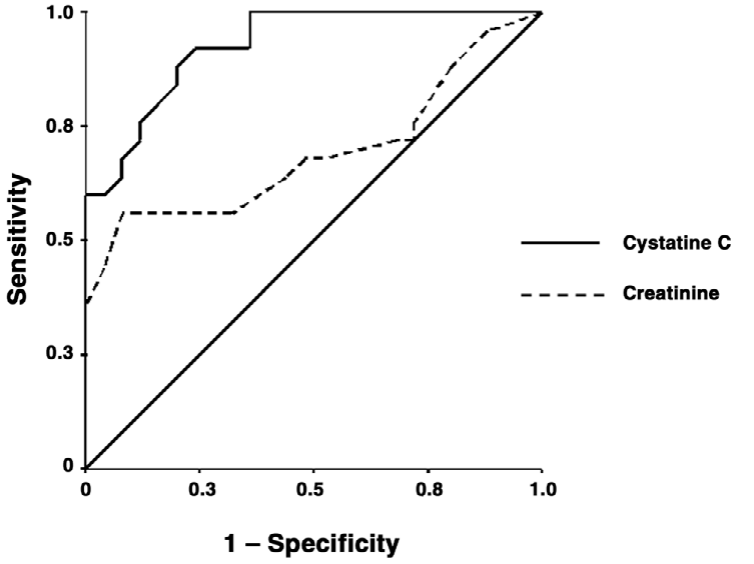
Nonparametric receiver operating characteristic plots of sensitivity and specificity of serum creatinine and cystatin C. Area under the curve (95% confidence interval): creatinine 0.694 (54.1–84.6) and cystatin C 0.927 (86.1–99.4).

Nonparametric receiver operating characteristic plots of sensitivity and specificity of serum creatinine and cystatin C for detecting renal dysfunction are shown in Fig. 3. The AUC for serum creatinine was 0.694 (95% confidence interval 54.1–84.6) and the AUC for serum cystatin C was 0.927 (95% confidence interval 86.1–99.4).

## Discussion

Monitoring renal function is extremely important in the management of critically ill patients. GFR, which can be measured by determining the clearance of various substances, is the 'gold standard' parameter for monitoring renal function. The ideal endogenous marker would be characterized by stable production rate, stable circulating levels (unaffected by pathological changes), lack of protein binding, free glomerular filtration, and lack of reabsorption or secretion; to date, no such marker has yet been identified. Some substances such as creatinine, urea, β_2_-microglobulin and retinol-binding protein have been used as endogenous markers of GFR, by measuring either their plasma levels or their renal clearance. Among them, the most useful markers for assessing GFR are serum creatinine and renal C_Cr_. This is secondary to their correlations with the renal clearance of some exogenous substances (inulin, creatinine-EDTA, iothalamate) that are considered 'gold standards' for determining GFR.

Creatinine production changes significantly according to the muscle mass of the body and dietetic factors. It is filtered by the glomeruli, but it is also secreted by the renal tubules. This tubular secretion contributes approximately 20% of the total creatinine excretion by the kidney, and it can increase as GFR decreases. All of these factors explain why serum creatinine concentration may not be a good parameter for accurate determination of GFR, especially at lower rates [1].

Cystatin C production in the body is a stable process that is not influenced by renal conditions, increased protein catabolism, or dietetic factors. Moreover, it does not change with age or muscle mass like creatinine does. Its biochemical characteristics allow free filtration in the renal glomerulus, and subsequent metabolism and reabsorption by the proximal tubule. For these reasons, serum cystatin C has been suggested to be an ideal endogenous marker of GFR [12-19].

Most studies conducted to evaluate whether there is a role for serum cystatin C in determining GFR involved measurement of the clearance of exogenous substances such as creatinine-EDTA [14,20-22], inulin [15,23], Tc-DTPA [24,25] and I-iothalamate [16,26,27]. Nevertheless, C_Cr _is still the most reliable marker for determining GFR on a routine basis, and multiple studies have used C_Cr _as a control for evaluating the role for serum cystatin C as a measure of GFR [28-30]. It is also a simple and cheap test, and, as mentioned above, its accuracy is sufficient for determining GFR. However, measurement of C_Cr _can yield erroneous findings in many situations, particularly when poor urine collection technique is employed. That the present study was conducted in critically ill individuals, all of whom had a bladder catheter in place, makes such errors less likely.

Previous studies [14-16,20-22,25,27] have found a wide range of correlations between 1/serum creatinine and clearance of exogenous substances (r = 0.50–0.89). In this study we found a correlation coefficient of 0.426 (*P *= 0.002) between C_Cr _and 1/serum creatinine. This difference in correlation rates among studies may be explained better by the characteristics of the patients than by the methods used. Most of the studies in the literature were performed in individuals who were in a stable clinical condition (healthy individuals, patients with various renal diseases, and oncological patients undergoing chemotherapy). GFR in critically ill individuals can change rapidly because of, for example, renal hypoperfusion secondary to shock or the use of nephrotoxic agents. Despite this, it is not uncommon to see changes in the serum creatinine for up to several days until the stabilization phase is reached. This may also explain the poor diagnostic usefulness of serum creatinine as seen in our study (AUC 0.694) compared with that in other studies [25]. Only five out of 25 (20%) of the individuals enrolled in our study who developed renal dysfunction exhibited high serum creatinine levels at the time when C_Cr _was tested. The delay that usually exists between the decline in GFR and that in serum creatinine makes the latter test poorly reliable for making therapeutic decisions in critically ill patients, such as a decision to change nephrotoxic agents or to increase renal perfusion.

We found a strong correlation between serum cystatin C concentrations and C_Cr _in this study (r = 0.832, *P *< 0.001). This is similar to findings reported by other investigators (r = 0.73–0.91) [14-16,20-22,25,27]. The diagnostic utility of cystatin C seen in our study (AUC = 0.927) is similar to that previously reported by other investigators [25]. The fact that most of our patients (76%) with acute renal dysfunction had high serum cystatin C levels at the time of C_Cr _evaluation demonstrates that cystatin C is a good marker for application in real time, and suggests that serum cystatin C is a better marker of GFR than is serum creatinine in unstable, critically ill patients (20% of patients with acute renal dysfunction had high serum cystatin C level).

## Conclusion

In the present study we evaluated and compared serum creatinine and serum cystatin C as markers of GFR in unstable, critically ill patients. Our data indicate that serum cystatin C is a good real-time marker of GFR in such patients. If this finding is subsequently confirmed, then the simplicity of serum cystatin C detection and its reasonable cost suggest that this test may soon replace C_Cr _as the biochemical marker of choice for monitoring GFR in a routine practice.

## Key messages

• 	In this study, serum cystatin C was found to be a good marker of GFR.

• 	Serum cystatin C was better at detecting changes in GFR than was serum creatinine in critically ill patients.

• 	Determination of serum cystatin C levels is useful in the management of critically ill patients.

## Abbreviations

AUC = area under the curve; C_Cr _= creatinine clearance; GFR = glomerular filtration rate.

## Competing interests

The author(s) declare that they have no competing interests.

## Authors' contributions

PV managed patients, recruited them into the study and participated in the drafting of the manuscript. MJ conceived the study and participated in its design and coordination. MCS and JM were subinvestigators of the study; their principal role was to recruit patients. PC analyzed the samples.

## Figures and Tables

**Table 1:** Demographic characteristics and clinical conditions of 50 critically ill patients at risk for developing acute renal dysfunction

Parameter	Details
Age (years; mean [range])	54 (21–86)
Sex (male/female)	34/16
APACHE II score (mean ± SD)	20 ± 9
Disease	
Multiple trauma	10/50 (20%)
Pulmonary disease	10/50 (20%)
Neurological disease	9/50 (18%)
Sepsis/septic shock	9/50 (18%)
Cardiological disease	7/50 (14%)
Postsurgical high risk	5/50 (10%)

APACHE, Acute Physiology and Chronic Health Evaluation; SD, standard deviation.
